# Comparative analysis of cancer vaccine settings for the selection of an effective protocol in mice

**DOI:** 10.1186/1479-5876-11-120

**Published:** 2013-05-12

**Authors:** Francesca Kalli, Rodolfo Machiorlatti, Florinda Battaglia, Alessia Parodi, Giuseppina Conteduca, Francesca Ferrera, Michele Proietti, Samuele Tardito, Marina Sanguineti, Enrico Millo, Daniela Fenoglio, Raffaele De Palma, Giorgio Inghirami, Gilberto Filaci

**Affiliations:** 1 Centre of Excellence for Biomedical Research, University of Genoa, Viale Benedetto XV n. 7, 16132, Genoa, Italy; 2 IRCCS Azienda Ospedaliero Universitaria San Martino IST, Genoa, Italy; 3 Department of Pathology and Center for Experimental Research and Medical Studies (CeRMS), University of Turin, Turin, Italy; 4 Department of Clinical and Experimental Medicine, Second University of Naples, c/o II Policlinico, Naples, Italy; 5 Department of Internal Medicine, University of Genoa, Genoa, Italy

## Abstract

**Background:**

Cancer vaccines are considered a promising therapeutic approach. However, their clinical results are not yet satisfactory. This may be due to the the difficulty of selection of an efficient tumor associated antigen (TAA) and immunization protocol. Indeed, the weak antigenicity of many TAA impairs the design of robust procedures, therefore a systematic analysis to identify the most efficient TAA is mandatory. Here, we performed a study to compare different gp100 vaccination strategies to identify the best strategy to provide a 100% protection against experimental melanoma in a reproducible manner.

**Methods:**

C57BL/6J mice were challenged subcutaneously with B16F10 melanoma cells, after vaccination with: a) mouse or human gp100_25-33_ peptide plus CpG adjuvant; b) mouse or human gp100 gene; c) mouse or human gp100_25-33_ peptide-pulsed dendritic cells (DC). Alternatively, a neutralizing anti-IL-10 monoclonal antibody (mAb) was subcutaneously administered at the site of tumor challenge to counteract regulatory cells. Finally, combinatorial treatment was performed associating human gp100_25-33_ peptide-pulsed DC vaccination with administration of the anti-IL-10 mAb.

**Results:**

Vaccination with human gp100_25-33_ peptide-pulsed DC was the most effective immunization protocol, although not achieving a full protection. Administration of the anti-IL-10 mAb showed also a remarkable protective effect, replicated in mice challenged with a different tumor, Anaplastic Large Cell Lymphoma. When immunization with gp100_25-33_ peptide-pulsed DC was associated with IL-10 counteraction, a 100% protective effect was consistently achieved. The analysis on the T-cell tumor infiltrates showed an increase of CD4+granzyme+ T-cells and a decreased number of CD4+CD25+Foxp3+ Treg elements from mice treated with either gp100_25-33_ peptide-pulsed DC vaccination or anti-IL-10 mAb administration. These data suggest that processes of intratumoral re-balance between effector and regulatory T cell subpopulations may play a critical protective role in immunotherapy protocols.

**Conclusions:**

Here we demonstrate that, in the setting of a cancer vaccine strategy, a comparative analysis of different personalized approaches may favour the unveiling of the most effective protocol. Moreover, our findings suggest that counteraction of IL-10 activity may be critical to revert the intratumoral environment promoting Treg polarization, thus increasing the effects of a vaccination against selected TAA.

## Background

Cancer immunotherapy is considered a promising therapeutic approach in oncology, and the recent successes obtained by Provenge and Ipilimumab support this view [1-3]. However, despite the discovery of a great number of tumor associated antigens (TAA) [4] and the setting of a large variety of immunotherapy protocols [5], their clinical efficacy remains dismal [4,6]. This is likely due to: (a) poor immunostimulatory efficacy of immunotherapy procedures, and (b) escaping mechanisms, as the accumulation of regulatory lymphocytes (Treg) within the tumor environment causing the impairment of anti-tumor cytotoxic cells [7]. Therefore, in presence of a wide array of TAA [8] and a variety of immunotherapy protocols [5], a systematic analysis leading to the identification of reference parameters for the selection and application of each single TAA is mandatory. This is the daunting challenging effort run by the NCI [8] and informative guidelines came from the CIC [9]. Thus, in the setting of an immunotherapy protocol using a specific TAA, a preliminary comparative analysis is recommended in order to identify the most immunogenic strategy able to achieve an optimal anti-tumor response.

Interleukin 10 (IL-10) is a pleiotropic cytokine secreted by a wide array of immune cells, including monocytes, macrophages, T cells, dendritic cells (DC), B cells, natural killer (NK) cells, mast cells, neutrophilic and eosinophilic granulocytes, and by several tumor cells [10,11]. This cytokine signals mainly via STAT3 regulating immunomodulating activities [12]. In particular, it decreases the antigen presenting activity of macrophages and DC mainly through HLA and co-stimulatory molecules down-regulation. Moreover, IL-10 suppresses the production of pro-inflammatory cytokines (i.e. IL-1α, IL-1β, TNF-α, IL-6, IL-8, IL-12, IL-18, granulocyte–macrophage colony-stimulating factor [GM-CSF], macrophage inflammatory protein-1, RANTES, leukemia inhibiting factor, IFNγ), and inhibits nuclear translocation of NF-kB [13,14]. At the same time, this cytokine is a NK cell activator [15] and a co-stimulator of mast cell proliferation [16,17]. Finally, IL-10 promotes B lymphocyte differentiation and immunoglobulin production [18], and plays a relevant role in immune regulation, mediating the activity of different regulatory T cell (Treg) subsets [19-21]. This pivotal role, associated with the finding that the tumor environment is usually rich in IL-10 secreted by tumor cells [22,23] and/or by tumor infiltrating elements [24], suggests that this cytokine has a critical function in tumor immune escaping. Thus, the inhibition of IL-10 has been proposed as a useful strategy in anti-cancer therapy [25,26].

In this study, we focused on a single TAA, gp100, in an established melanoma model, analyzing different gp-100 centred vaccination strategies. Our main aim was to identify a protocol able to consistently provide 100% tumor protection, and this aim was achieved only when the most efficacious effector strategy was associated with IL-10 blockade to counteract Treg activity.

## Materials and methods

### Cell lines

The cell lines used in this study were: a) B16F10 melanoma cells (a spontaneous C57BL/6J-derived melanoma widely used for the evaluation of therapy [27]); b) RMA/S cells, a murine T-lymphoma cell line deficient in the presentation of endogenously synthesized antigens by MHC class I molecules [28] (kindly provided by Dr. G. Pietra from the Advanced Biotechnology Center of Genoa); c) VAC cell line, which is an immortalized line from primary lymphoma derived from a NPMALK–transgenic mouse backcrossed onto the BALB/c background (CD4+/ALK+ lymphoblastic lymphoma) [29], and used for generating syngenic s.c. tumorgrafts of Anaplastic Large Cell Lymphoma (ALCL).

All lines were cultured in RPMI 1640 medium (GIBCO, Life Technologies Ltd., Paisley, UK) containing 10% heat-inactivated fetal bovine serum (FCS, Euroclone, Wetherby, UK), 2 mM/L L-glutamine, 100 U/ml penicillin, 100 μg/ml streptomycin (GIBCO) in a humidified atmosphere at 37°C and 5% CO_2_.

### Mice

C57BL/6J, 6- to 8-week old, female mice were purchased from Harlan Laboratories (S.Pietro al Natisone, Udine, Italy). BALB/c mice were purchased from Charles River Laboratories (Calco, Lecco, Italy).

Animal procedures were conducted in accordance with the institutional guidelines and experiments have been reviewed and approved by Ethics Committee for Experimentation on Animals (CSEA) of Genoa and Turin.

### Synthesis of the highly immunogenic peptide encompassing residues 25–33 of mouse and human gp100

Mouse (EGSRNQDWL) and human (KVPRNQDWL) gp100_25–33_ peptides, as well as influenza virus nucleoprotein A NP_366-374_ (ASNENMETM) peptide, were in house synthesized at the biochemical facility of the Centre of Excellence for Biomedical Research by standard methods of solid phase peptide synthesis, which follows a 9-fluorenylmethoxycarbonyl (Fmoc) strategy with minor modifications [30]. Synthesized compounds were purified by reverse-phase high performance liquid chromatography (HPLC) and molecular weights confirmed by electrospray ion-trap mass spectrometry. The purification of individual compounds was obtained on a Shimadzu LC-9A preparative HPLC (Shimadzu, Kyoto, Japan) equipped with a Waters C18 μBondapack column (19 x 300 mm; Waters, Milford, MA).

### Culture of bone marrow derived DC and activation for the expression of gp100 on its surface

Bone marrow (BM) derived DC were generated as described [31]. BM cells were harvested from thigh-bones and tibias of naïve C57BL/6J mice and washed with phosphate-buffered saline (PBS) (Invitrogen, Life Technologies). Cells (1x10^6^/ml) were suspended in DC medium consisting in RPMI 1640 supplemented with 10% heat-inactivated FCS (HyClone, Logan, UT), 2 mM L-glutamine, 100 U/ml penicillin, 100 μg/ml streptomycin, 100 mM sodium pyruvate, 10 mM Hepes buffer solution, 100 mM non essential amino acids solution, 50 μM β-mercaptoetanol (GIBCO), and plated plate with 20 ng/ml murine GM-CSF (6 well plates, PeproTech, Rocky Hill, NJ). On day 3, fresh medium was added plus 5 ng/ml murine GM-CSF (PeproTech). On day 7, the DC phenotype was checked using the following mAbs: fluorescein isothiocyanate (FITC) conjugated anti-MHC class II (HLA DR), phycoerythrin (Pe) cyanin 7 conjugated anti-CD11c, allophycocyanin (APC) conjugated anti-CD86 (Biolegend, San Diego, CA). More than 90% of the cells showed high expression of all DC markers. Before administering DC to animals, DC (1x10^6^/ml) were activated with CpG (1 μg/ml) for 8 hours; then, activated DC (2x10^6^/ml) were pulsed by incubation with mouse or human gp100_25-33_ peptide (2 μM) at 37°C for 2 hours.

### gp100 vaccination of C57BL/6J mice against B16F10 melanoma

In vivo experiments were performed analyzing at least 5 animals per group; each experiment was repeated for 3 times.

C57BL/6J mice were immunized against gp100 molecule following three different protocols: 1) peptide plus adjuvant; 2) gene immunization; 3) peptide-pulsed DC immunization (summarized in Additional file 1: Figure S1 in Additional files). *Peptides plus adjuvant protocol*: peptides were administered by injecting subcutaneously either mouse (mgp100_25-33_) or human (hgp100_25-33_) gp100_25-33_ preparations (100 μg/mouse) in association with CpG (30 μg/mouse), as adjuvant. The unrelated NP_366-374_ peptide from influenza virus nucleprotein A was used as negative control in preliminary experiments. *Gene immunization*: this protocol was executed administering intramuscularly plasmids expressing either mouse or human gp100 gene sequence (100 μg/mouse). A pCMVscript plasmid (Stratagene) was used as gene vector. The gene coding for mouse gp100 molecule was amplified from cDNA obtained after extraction of RNAs from B16F10 melanoma cells. The construct coding for human gp100 was kindly provided by Prof. N. Restifo (NIH, Bethesda). PCR amplified products were first cloned into a sequencing vector (TOPO TA cloning from Invitrogen) and then Sanger sequenced. Both mouse and human gp100 cloned genes were subsequently transferred in the eukaryotic expressing vector pCMVscript.The empty pCMVscript plasmid was used as negative control in preliminary experiments. *Peptide-pulsed DC immunization*: this protocol was performed injecting in the right flank DC (2x10^6^/mouse) pulsed with either mouse or human gp100_25-33_ peptide. Unpulsed DC or DC pulsed with the unrelated NP_366-374_ peptide were used as negative control in preliminary experiments. The schedule of each protocol consisted in three immunizations, administered weekly. At the time of the third immunization, mice received the tumor challenge, that consisted in sc. injection in the controlateral limb of B16F10 cells (1x10^5^cells/mouse). Tumor masses were measured with calipers at 2–3 days intervals by measuring long and short axes. Area was calculated according to the formula: tumor area = length × width in mm^2^. Mice were sacrificed when tumors reached >100 mm^2^ or when ulceration and/or bleeding developed.

### Immunotherapy of B16F10 melanoma in C57BL/6J mice or ALCL in BALB/c mice based on IL-10 neutralization

In vivo experiments were performed analyzing 5 animals per group; each experiment has been repeated 3 times.

C57BL/6J were s.c. challenged with B16F10 melanoma (1x10^5^ cells/mouse) while BALB/c mice were injected s.c. withVAC (1x10^6^ cells/mouse) cells. The neutralizing anti-IL-10 (Anti-Mouse IL-10 Functional Grade Purified, clone JES5-16E3) (eBioscience, San Diego, CA) mAb (150 μg/mouse) was administered subcutaneously at the site of tumor challenge immediately after tumor cell injection as well as after one and two weeks from baseline. A rat IgG2b,k control isotypic antibody (Biolegend, San Diego, CA) was administered using the identical schedule adopted for the anti-IL10 mAb. When IL-10 blockade was associated in combinatorial treatment to gp100 vaccination, the neutralizing anti-IL-10 mAb (150 μg/mouse), or its isotypic control, were subcutaneously administered as described above.

### Purification of splenocytes and intratumor lymphocytes

Spleens or tumors, removed from sacrificed mice, were minced with a sharp sterile blade, placed in a 40-μm nylon cell strainer (BD Biosciences, Franklin Lakes, NJ), and pressed with the plunger of a syringe until cellular elements were released. Red blood cells were lysed with red blood cell lysing buffer (Sygma Aldrich), and washed. Tumor or splenocyte suspensions were collected in RPMI 1640 medium supplemented with 10% FCS. Splenocytes from treated and untreated mice (2.5x10^6^/ml) were plated in flasks in the presence of irradiated (3000 rad) splenocytes (1x10^6^/ml) and gp100_25–33_ peptide (10 μg/ml) and cultured for 5 days. After incubation, splenocytes were collected and separated by density gradient (Biochrom AG, Berlin, Germany). Splenocyte samples were used for phenotypical analysis, the remaining portion for ELISPOT assay.

In the ALCL model, we applied two overnight incubations with ALK inhibitor CEP28122 (20 ng/ml) followed by separation by density gradient before phenotype analysis.

### Immunofluorescence and flow cytometric (FACS) analysis

Phenotypes of tumor infiltrating T lymphocytes (TIL) and whole splenocytes were analyzed by immunofluorescence incubating the cells (1×10^5^ lymphocytes in 100 μl of PBS) with specific mAbs at 4°C for 30 minutes in the dark. The following mAbs were used: FITC conjugated anti-GranzymeB (eBioscience), Pe-conjugated anti-Foxp3 (eBioscience), Peridinin chlorophyl protein (PerCP)-cyanin 5.5-conjugated anti-CD8 (Biolegend), anti-CD28(Biolegend), PeCy 7-conjugated anti-CD3(BD Pharmingen), APC-conjugated anti- (BD Pharmingen), APCCy 7-conjugated anti-CD4 (BD Pharmingen). For intracellular staining, cells were permeabilized and fixed using FOXP3 Fix/Perm Buffer Set (Biolegend) according to manufacturer’s instructions and then incubated with fluorochrome conjugated anti-GranzymeB and anti-Foxp3mAb; fluorochrome-conjugated isotype matched Abs were also used as controls. After the staining, the analysis was performed by flow cytometry using a FACSCanto II flow cytometer equipped with FACS Diva software (BD).

### Elispot assay

Detection of IFNγ production by splenocytes in response to peptide stimulation was carried out using an enzyme-linked immunospot (Elispot) assay (Millipore, Merck KGaA, Darmstadt, Germany) according to the manufacturer’s instructions. Briefly, splenocytes (2x10^5^/well) from treated and untreated mice, cultured for 5 days with irradiated splenocytes plus peptide (10 μg/ml) were harvested, and dead cells were removed by centrifugation on density gradient (Biochrom). Then, cells were collected and washed before incubating them overnight at 37°C with either mgp100_25-33_- or hgp100_25-33_-pulsed RMA/S cells (1x10^4^ cells) in 96-well plates coated with 10 μg/ml of unlabeled anti-mouse IFNγ rat monoclonal capture antibody (clone AN18, Millipore). At the end of incubation, a biotinylated detection antibody (2 μg/ml) was added to the wells and reacted with alkaline phosphatase-streptavidin (100 μl/well) and the development with BCIP/NBT Phosphatase substrate (50 μl/well) (KPL, Kirkegaard & Perry Laboratories, Inc.). Frequencies of IFNγ producing cells were measured using a BioReader 3000 Elispot Reader (Bio-Sys GmbH). Data are expressed as the mean number of spots per duplicate.

### Proliferation inhibition assay

The suppression activity was evaluated by monitoring the proliferation of splenocytes isolated from C57BL/6J and from BALB/c mice by dye dilution approach. For this assay, splenocytes (used as responder cells) were stained with carboxyfluorescein succinimidyl ester (CFSE) 5 μM (Molecular Probes, Life Technologies) and co-cultured with Concanavalin A (Con A) (Sigma Aldrich) at 5 μg/ml for 5 days in a 96 well flat bottomed plate with or without tumor infiltrating lymphocytes (TIL) from melanoma or lymphoma tumors (used as suppressor cells). Splenocytes:TIL cell ratio was 2:1. Co-cultures with TIL from both tumors were also performed in the presence or absence of the neutralizing anti-IL-10 mAb (clone JES5-16E3 eBioscience) (10 μg/ml) or of its isotypic control. After 5 days the cultures were washed in PBS and acquired by FACS Canto flow cytometer equipped with FACS Diva software (Becton Dickinson, BD). A total of 50000 CFSE positive responder cells were analyzed. The results were expressed as percentage of CFSE bright splenocytes.

### Immuno-histochemical analysis

Tumors specimens were taken from either anti-IL-10 mAb treated mice or controls and fixed with nitrogen vapors. Tissue sections, obtained from melanoma specimens, were stained with monoclonal antibody against IL-10 (Purified Rat anti-mouse IL-10, clone JES5-16E3, BD, Milano, Italy) following the manufacturers’ instructions.

### Statistical analysis

Comparisons between mean values were performed by unpaired or paired t test using the Graph-Pad Prism 5.0 software (Graph-Pad Software, Inc, San Diego, CA, USA). Data were considered statistically significant when *p* ≤ 0.05.

## Results

### Comparative analysis of protective effect among protocols inducing anti-cancer effector immune responses

Our studies wanted to compare different strategies of vaccination. Since each of these strategies would require specific controls, we preliminarly performed an analysis of the effects of each control on tumor growth. The controls were: a) s.c. immunization with the NP_147-155_ gp100 unrelated peptide plus CpG adjuvant; b) i.m. administration of the empty pCMVscript plasmid; c) s.c. administration of DC unpulsed or pulsed with the NP_147-155_ gp100 unrelated peptide. Additional 1: FigureS2 shows that in repeated experiments none of these procedures significantly modified tumor growth. Based on these data, in order to have a unique, homogeneous control for all the experiments we performed the following studies using spontaneous tumor growth as universal control.

**Figure 1:**
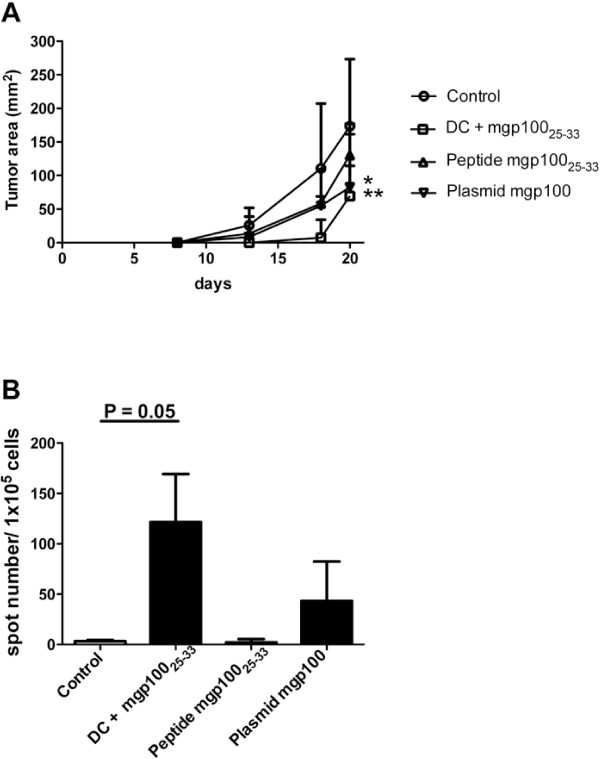
**Comparative analysis of the different gp100 vaccination protocols in a syngeneic setting.** (**A**) B16F10 melanoma growth curves in differently treated and control mice (*p = 0.05; **p = 0.01); (**B**) Elispot analysis of frequency of IFNγ-secreting splenocytes from differently treated and control mice.

In a syngeneic setting gp100_25-33_ peptide-pulsed DC vaccination was the most effective, inducing >50% tumor mass reduction, while peptide vaccination and gene vaccination had comparable efficacies (Figure 1A). According to the protection results, mice treated with peptide-pulsed DC had among splenocytes a significantly higher frequency of IFNγ-secreting T cells in response to gp100_25-33_ peptide stimulation than control animals (Figure 1B), as assessed by Elispot analysis.

**Figure 2:**
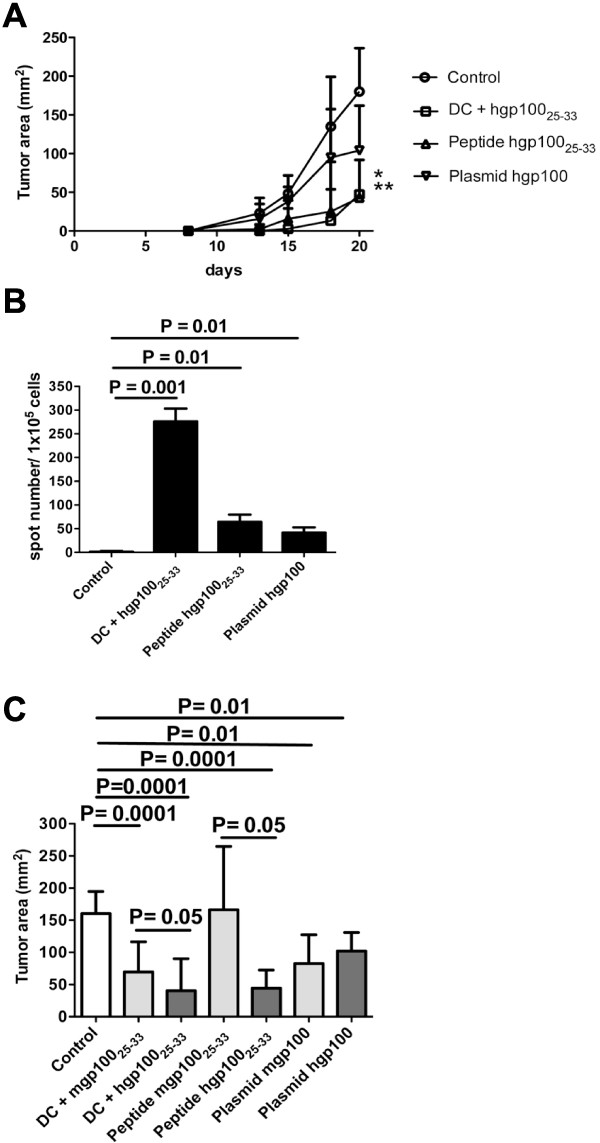
**Comparison of gp100 vaccination protocols performed either in syngeneic or xenogeneic setting.** (**A**) Comparative analysis of the different gp100 vaccination protocols in a xenogeneic setting. B16F10 melanoma growth curves in differently treated and control mice (*p = 0.05; **p = 0.01); (**B**) Elispot analysis of frequency of IFNγ-secreting splenocytes from differently treated and control mice in a xenogeneic setting; (**C**) Comparison of gp100 vaccination protocols performed in syngeneic (light grey columns) or xenogeneic (dark grey columns) settings. Data refer to the assessment performed after 21 days from B16F10 melanoma challenge when mice were sacrificed.

These experiments were repeated in a xenogeneic setting, using the human gp100_25-33_ peptide or a plasmid coding the human gp100 protein. In this setting, vaccination with DC pulsed with the gp100_25-33_ peptide remained the most effective protocol, although peptide plus adjuvant vaccination achieved comparable results (Figure 2A).

The Elispot analysis of frequency of IFNγ-secreting T splenocytes in response to gp100_25-33_ peptide showed a higher number of positive cells versus controls, in all groups of treated mice (Figure 2B).

These data clearly indicate that the protocol with gp100_25-33_ peptide-pulsed DC was the most effective strategy. To verify which protocol between the syngeneic and the xenogeneic setting could produce the most protective action against tumor growth, we combined all data and compared them. Figure 2C shows that vaccination performed with the human gp100_25-33_ peptide was able to reduce melanoma growth more than the vaccination performed with the mouse corresponding peptide.

### Analysis of effector and regulatory T cells in mice vaccinated with gp100_25-33_ peptide-pulsed DC

**Figure 3:**
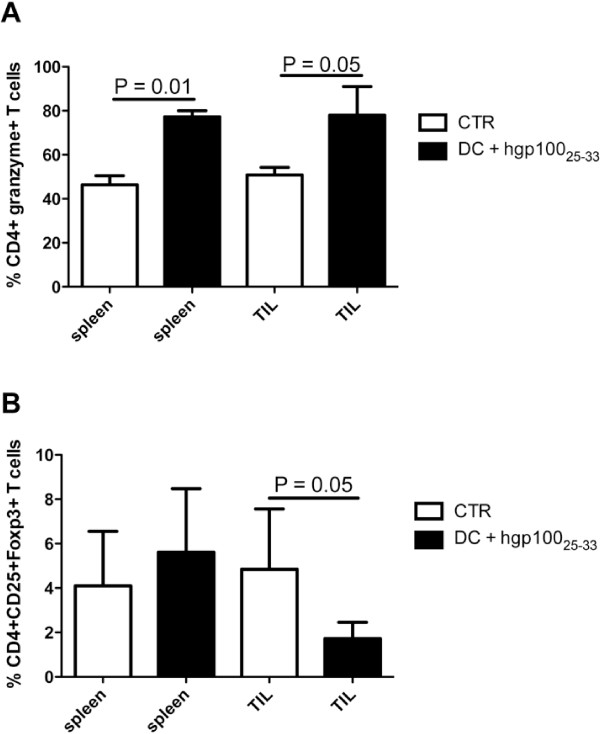
**Frequency of CD4+granzyme+ and CD4+CD25+Foxp3+ T lymphocytes in vaccinated mice.** FACS analyses of frequency of CD4+granzyme+ (**A**) and CD4+CD25+Foxp3+ (**B**) T lymphocytes were performed in mice immunized or not with gp100_25-33_ peptide-pulsed DC. The analyses were executed on splenocytes and TIL from immunized (black columns) or untreated (white columns) mice. Data in both panels are expressed as percentage of positive cells out of the total CD3+ T cell population.

To understand the cellular mechanisms underlying the effects of pulsed DC vaccination, the frequencies of CD4+granzyme+ and CD8+granzyme+ T cells (representative of effector T cell populations) and of CD4+CD25+FoxP3+ Treg (representative of regulatory lymphocytes) were analyzed in spleens and in tumors from treated and untreated mice. Figure 3A shows that the frequency of CD4+granzyme+ T cells in treated mice was significantly higher than in controls both in spleens and in the host tumor microenvironment. No significant variations were observed in the CD8+granzyme+ T cell compartment (not shown). Moreover, a decrease of CD4+CD25+Foxp3+ Treg was observed in treated mice with respect to untreated controls in the tumors but not in the spleen (Figure 3B).

### Protective effect of IL-10 neutralization against melanoma and ALCL growth

**Figure 4:**
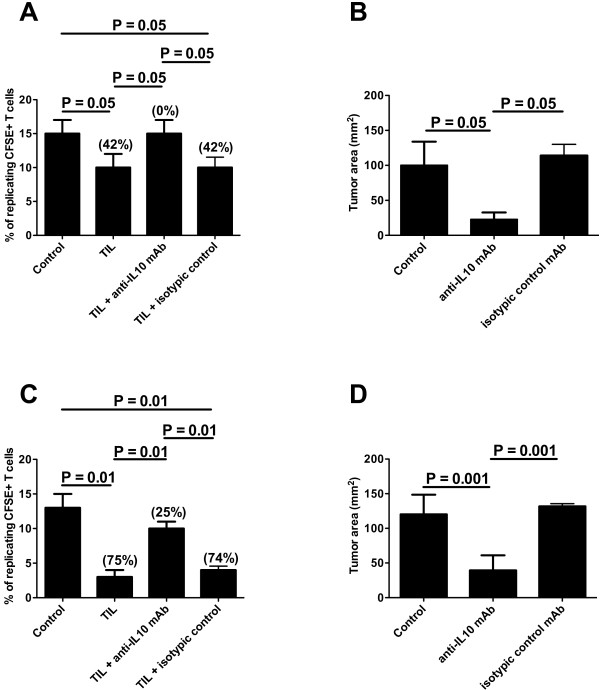
**Involvement of IL-10 in tumor growth.** (**A** and **C**) Suppression assay performed with TIL purified from melanoma (**A**) or ALCL (**C**). CFSE-labeled syngeneic splenocytes were in vitro stimulated with Con A and co-incubated with TIL in the presence or not of an anti-IL-10 mAb or its relative isotypic control. Data are expressed as percentage of replicating, CFSE+ cells; percentage of inhibition are indicated in parentheses. (**B** and **D**) Mean tumor dimensions in mice challenged after 21 days with either B16F10 melanoma (**B**) or VAC ALCL (**D**) cells and treated or not with an anti-IL-10 mAb or its relative isotypic control.

Since none of the immunization protocols exerted a complete protection against B16F10 melanoma engrafting, we hypothesized that a specific counteraction of the regulatory T cell compartment could increase the efficiency of spontaneous and/or vaccine induced anti-tumor effector mechanisms. This hypothesis was supported by the data generated using Ipilimumab in human protocols [1,32]. Indeed, the observation that TIL purified from melanoma lesions exerted a regulatory activity in an IL-10 dependent fashion confirmed this idea (Figure 4A). Moreover, melanoma sections stained with an anti-IL-10 mAb demonstrated the presence of the cytokine in the tumor microenvironment (data not shown).

Hence, we decided to verify whether in vivo IL-10 neutralization could represent a useful mean for counteracting the activity of the regulatory T cell compartment.

Indeed, IL-10 blockade by an anti-IL-10 mAb, administered in monotherapy at the site of tumor challenge, led to a significant delay in melanoma growth, reminiscent of what observed in different experimental settings [33] (Figure 4B). Importantly, the same anti-IL-10 mAb added in vitro (20 μg/ml) to B16/F10 melanoma cell culture did not change either cell proliferation rate or cell viability, ruling out the possibility of any direct toxic effect (not shown).

In order to understand whether the effects mediated by IL-10 neutralization were melanoma-specific or could be replicated in a different unrelated type of tumor, the same experiment was performed using mouse anaplastic large cell lymphoma (ALCL) cells (ALK+ VAC cells, [29]) as challenging tumor. Preliminarly, the IL-10-dependent regulatory activity exerted by TIL was also demonstrated for this model (Figure 4C). Indeed, in vivo IL-10 blockade resulted in a significant protection against ALCL growth (Figure 4D).

**Figure 5:**
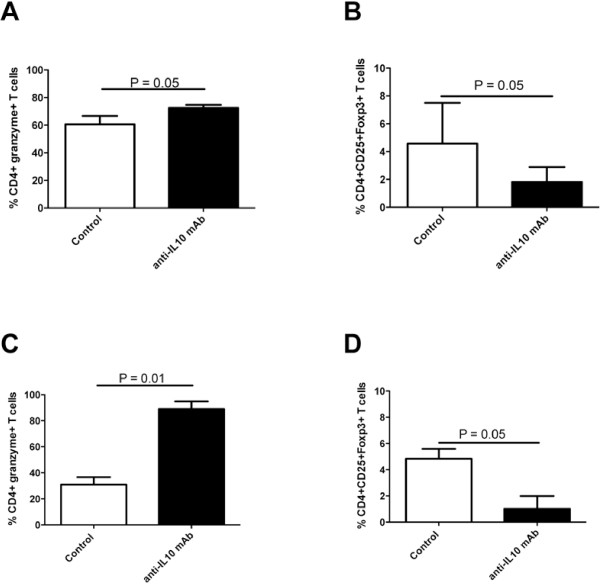
**Frequency of CD4+granzyme+ and CD4+CD25+Foxp3+ T lymphocytes in mice treated with an anti-IL-10 mAb.** FACS analyses of frequency of CD4+granzyme+ (**A** and **C**) and CD4+CD25+Foxp3+ (**B** and **D**) T lymphocytes were performed in mice untreated or treated with an anti-IL-10 mAb. The analyses were executed on TIL from treated (black columns) or untreated (white columns) mice. (**A**) and (**B**) panels refer to experiments performed in C57BL/6J mice challenged with B16F10 melanoma cells; (**C**) and (**D**) panels refer to experiments performed in Balb/c mice challenged with VAC ALCL cells. In all panels, data are expressed as percentage of positive cells out of the total CD3+ T cell population.

In the attempt to understand the mechanisms responsible for the protective effect of IL-10 blockade, phenotypic analyses were performed on TIL of mice treated with the neutralizing anti-IL-10 mAb. These analyses showed that in both tumor microenvironments, IL-10 treatment caused a significant increase of CD4+granzyme+ T cells with a concomitant decrease of CD4+CD25+FoxP3+ Treg, reminiscent of what observed after vaccination with gp100_25-33_ peptide-pulsed DC (Figure 5). No changes in the percentage of either CD4+granzyme+ T cells or CD4+CD25+FoxP3+ Treg were observed in splenocytes when we compared treated and untreated mice (not shown).

**Figure 6:**
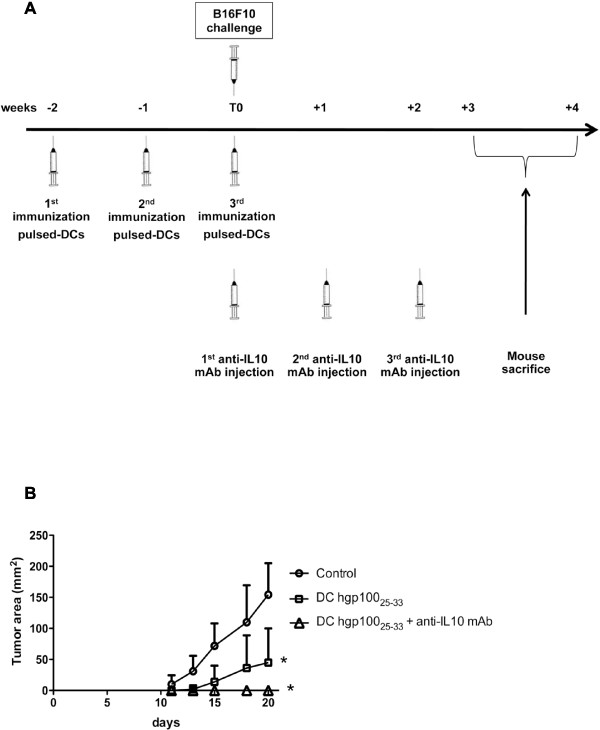
**Combinatorial treatment of B16F10 melanoma: vaccination with gp100**_**25-33 **_**pulsed-DC plus neutralizing anti-IL-10 mAb administration.** (**A**) Schedule of treatment; (**B**) B16F10 melanoma growth curves in differently treated and control C57BL/6J mice (*p = 0.05).

Based on these results, we arrived to the conclusion that a combinatorial treatment associating Treg counteraction, through IL-10 blockade, with stimulation of the anti-tumor immune response, through vaccination, could have addictive/synergic efficacy. Accordingly, the combinatorial administration of the anti-IL10 mAb, but not its related isotypic control (not shown), to gp100_25-33_ peptide-pulsed DC vaccinated mice led to a complete protection from B16F10 melanoma growth (Figure 6).

## Discussion

Our data demonstrate that: a) the vaccination with DC pulsed with gp100_25-33_ peptide was the most effective in protecting C57BL/6 mice from B16F10 melanoma development compared to other gp100 vaccination protocols such as peptide plus adjuvant or gene vaccination; b) different protective effects were observed in syngeneic or xenogeneic settings in gp100 peptide-centred cancer vaccination protocols; c) the protective effects exerted by DC vaccination were related to increased intratumoral concentration of granzyme+ CD4+ T cells and decreased concentration of CD4+CD25+Foxp3+ Treg; d) counteraction of IL-10 by a neutralizing mAb lowered the rate of tumor development, inducing an increase of intratumoral concentration of granzyme+ CD4+ T cells and a decrease of CD4+CD25+Foxp3+ Treg; e) only the combinatorial treatment associating DC vaccination with IL-10 blockade was able to abrogate completely tumor growth.

Cancer vaccination is based on the identification at the molecular level of antigens expressed preferentially by specific tumors. Although Sipuleucel-T is the first cancer vaccine that recently reached the clinic after receiving the authorization for clinical use in prostate cancer [34], it is believed that immunological protocols will reach a full clinical efficacy and wide application in oncology only when well defined oncoantigens will be proved efficient by in vivo protective experiments. Hence, this process requires a systematic analysis of the protective effect of the different TAA and related protocols of administration, since several variables, including type of antigen, route of administration, molecular form of antigen administration, selection of adjuvants, may condition the outcome of the treatment [35]. In this paper, we compare the protective efficacy of different protocols of cancer vaccination based on a classical, widely adopted model system constituted by gp100 vaccination in B16F10 melanoma [27]. Intriguingly, the comparison of different vaccination protocols, such as peptide plus adjuvant, gene immunization and peptide-pulsed DC immunization, showed different levels of protective efficacy among the three protocols, being DC vaccination the most effective one. This is a remarkable observation since suggests that adequate stimulation of a single (or limited number of) T cell clone(s), specific for one specific TAA epitope, as induced by peptide-pulsed DC vaccination, may have more pronounced protective effects than multi-epitope mediated stimulation of a wide TAA-specific T cell repertoire, as induced by vaccination with a gene coding for the entire TAA molecule. Importantly, the protective effects of identical protocols performed in syngeneic or xenogeneic settings differed greatly. This is not surprising since the efficacy of xenogeneic immunization has been already reported in different models of experimental tumor and associated with the expression of a heteroclitic epitope by the immunizing agent [36,37]. The use of xenogeneic or modified tumor antigens, as a system for increasing the immunogenicity of the vaccine and breaking tolerance, has been suggested for long time [4,38]. However recent evidences from human trials seems to disprove the efficacy of this approach [39]. Taken together, data reported here further demonstrate the need of experimental testing to assess the most efficient protocols. Indeed, our analyses deserve to be replicated in different tumor model systems in order to verify whether our findings represent a generalized phenomenon in cancer vaccination.

The protective effect exerted by a cancer vaccination protocol is likely related to a number of biological factors concerning the immunogenicity of the antigen, the competence of the T and B cell repertoires, the level of activation of innate immunity, the capacity of the antigen to elicit immune responses involving a wide scenario of lymphocyte subtypes (T helper, CTL, Th17, B lymphocytes) and, last but not least, the number of boosts [40-44]. Hence, monitoring cancer vaccine-induced immune response, should take into consideration a variety of factors, likely becoming an impossible challenge for clinical trials.

Trying to simplify what underlies complex biological networks, the outcome of an immune response may be function of the ratio between the effector and the regulatory response [45-47]. In this study, we selected the intratumoral concentration of granzyme+CD4+ or CD8+ T cells as a parameter representative of effector immune responses. Similarly, we analyzed intratumoral concentration of CD4+CD25+FoxP3+ Treg as a parameter representative for regulatory circuits. Strikingly, protective effects induced by the different protocols of cancer vaccination were related and dependent on increasing number of tumor infiltrating CD4+granzyme+ T cells and decreasing concentration of Treg. Whether the effector/regulatory ratio can be further exploited to assess the efficacy of a cancer vaccine-induced immune response needs to be analyzed in future studies.

Notwithstanding the clear protective efficacy of peptide-pulsed DC vaccination, this protocol was not able to provide abrogation of tumor growth in 100% of treated mice. This prompted us to test an alternative immunotherapy based on IL-10 counteraction. This choice derives from our previous observation related to the presence within tumors of IL-10-secreting Treg able to strongly impair anti-tumor immune response [21]. Since presence of both IL-10 and IL-10-secreting Treg was demonstrated in B16F10 melanoma [our data and 24], we explored the possibility to counteract the effects of intratumoral IL-10 to unleash protective effector responses. In fact, the administration of a neutralizing anti-IL-10 mAb exerted protective effects similar to that demonstrated by peptide-pulsed DC vaccination, inducing intratumoral recruitment of CD4+granzyme+ T cells and deplenishing of CD4+CD25+FoxP3+ Treg. These effects were not specific for B16F10 melanoma and they could be replicated in the unrelated cancer model of ALCL. More importantly, when peptide-pulsed DC vaccination and IL-10 blockade were applied in a combinatorial protocol, complete abrogation of tumor growth was achieved in 100% of treated mice.

## Conclusions

Taken together, our data show that, in order to improve vaccine efficacy, comparative experimental testing of the relative protective effects of different protocols of vaccination should be performed before translating the immunotherapy to humans. The usage of IL-10 blockade represents a useful strategy for successful treatment of cancer in preclinical settings. These findings encourage the exploitation of this strategy in human settings.

## Competing interests

The authors have no competing interests to disclosure.

## Author’s contributions

KF performed in vivo and in vitro experiments and drafted the manuscript, MR carried out immunization based on IL-10 neutralization in ALCL tumor model, BF performed in vivo and in vitro experiments, PA and FD carried outflow cytometric analysis, CG, FF and TS participated in experiments performed with plasmids, PM performed in vivo and in vitro experiments, SM performed tumor implantation, ME synthesized peptides, DPR carried out immunohistochemical analysis and participated in writing the paper, IG participated in designing the experimental flow chart and supervised the results, FG participated in designing the experimental flow chart, supervised the results and wrote the paper. All authors read and approved the final manuscript.

## Supplementary Material

Additional file 1 Includes the Supplementary Figure 1 and the Supplementary Figure 2. Click here for file

## References

[B1] Kwek SS Cha E Fong L Unmasking the immune recognition of prostate cancer with CTLA4 blockade Nat Rev Cancer 2012 12 289 297 10.1038/nrc3223 22378189 PMC3433280

[B2] Kantoff PW Higano CS Shore ND Berger ER Small EJ Penson DF Redfern CH Ferrari AC Dreicer R Sims RB Xu Y Frohlich MW Schellhammer PF Sipuleucel-T immunotherapy for castration-resistant prostate cancer N Engl J Med 2010 363 411 422 10.1056/NEJMoa1001294 20818862

[B3] Farolfi A Ridolfi L Guidoboni M Nicoletti SVL Piciucchi S Valmorri L Costantini M Scarpi E Amadori D Ridolfi R Ipilimumab in advanced melanoma: reports of long-lasting responses Melanoma Res 2012 22 263 270 10.1097/CMR.0b013e328353e65c 22516968

[B4] Parmiani G Castelli C Dalerba P Mortarini R Rivoltini L Marincola FM Anichini A Cancer immunotherapy with peptide-based vaccines: what have we achieved? Where are we going? J Natl Cancer Inst 2002 94 Suppl 11 805 818 12048268 10.1093/jnci/94.11.805

[B5] Kirkwood JM Butterfield LH Tarhini AA Zarour H Kalinski P Ferrone S Immunotherapy of cancer in 2012 CA Cancer J Clin 2012 62 Suppl 5 309 335 22576456 10.3322/caac.20132 PMC3445708

[B6] Stan R Wolchok JD Cohen AD DNA vaccines against cancer Hematol Oncol Clin North Am 2006 20 613 636 10.1016/j.hoc.2006.02.004 16762727

[B7] Borghaei H Smith MR Campbell KS Immunotherapy of cancer Eur J Pharmacol 2009 625 41 54 10.1016/j.ejphar.2009.09.067 19837059 PMC2783916

[B8] Cheever MA Allison JP Ferris AS Finn OJ Hastings BM Hecht TT Mellman I Prindiville SA Viner JL Weiner LM Matrisian LM The prioritization of cancer antigens: a national cancer institute pilot project for the acceleration of translational research Clin Cancer Res 2009 15 5323 5337 10.1158/1078-0432.CCR-09-0737 19723653 PMC5779623

[B9] Hoos A Britten C The immuno-oncology framework. Enabling a new era of cancer therapy Oncoimmunology 2012 1 Suppl 3 334 339 22737609 10.4161/onci.19268 PMC3382871

[B10] Sabat R IL-10 family of cytokines Cytokine Growth Factor Rev 2010 21 315 324 10.1016/j.cytogfr.2010.11.001 21112807

[B11] Sato T McCue P Masuoka K Salwen S Lattime EC Mastrangelo MJ Berd D Interleukin 10 production by human melanoma Clin Cancer Res 1996 2 1383 1390 9816311

[B12] Liu X Li J Zhang J STAT3-Decoy ODN inhibits cytokine autocrine of murine tumor cells Cell Mol Immunol 2007 4 Suppl 4 309 313 17764622

[B13] Mosser DM Zhang X Interleukin-10: new perspectives on an old cytokine Immunol Rev 2008 226 205 218 10.1111/j.1600-065X.2008.00706.x 19161426 PMC2724982

[B14] Hamidullah Changkija B Konwar R Role of interleukin-10 in breast cancer Breast Cancer Res Treat 2012 133 11 21 10.1007/s10549-011-1855-x 22057973

[B15] Alas S Emmanouilides C Bonavida B Inhibition of interleukin 10 by rituximab results in down-regulation of Bcl-2 and sensitization of B-cell non-Hodgkin’s lymphoma to apoptosis Clin Cancer Res 2001 7 709 723 11297268

[B16] Fitzgerald KA O’Neill LAJ Gearing AJH Callard RE The cytokine factsbook 2001 2 London: Academic

[B17] Haddad JJ Saade´ NE Safieh-Garabedian B Interleukin-10 and the regulation of mitogen-activated protein kinases: are these signalling modules targets for the anti-inflammatory action of this cytokine? Cell Signal 2003 15 255 267 10.1016/S0898-6568(02)00075-X 12531424

[B18] Fluckiger AC Garrone P Durand I Galizzi JP Banchereau J Interleukin 10 (IL-10) upregulates functional high affinity IL-2 receptors on normal and leukemic B lymphocytes J Exp Med 1993 178 1473 1481 10.1084/jem.178.5.1473 8228801 PMC2191252

[B19] Hall BM Verma ND Tran GT Hodgkinson SJ Distinct regulatory CD4+T cell subsets; differences between naїve and antigen specific T regulatory cells Curr Opin Immunol 2011 23 641 647 10.1016/j.coi.2011.07.012 21840184

[B20] Filaci G Fravega M Negrini S Procopio F Fenoglio D Rizzi M Brenci S Contini P Olive D Ghio M Setti M Accolla RS Puppo F Indiveri F Nonantigen specific CD8+ T suppressor lymphocytes originate from CD8+CD28- T cells and inhibit both T-cell proliferation and CTL function Hum Immunol 2004 65 142 156 10.1016/j.humimm.2003.12.001 14969769

[B21] Filaci G Fenoglio D Fravega M Ansaldo G Borgonovo G Traverso P Villaggio B Ferrera A Kunkl A Rizzi M Ferrera F Balestra P Ghio M Contini P Setti M Olive D Azzarone B Carmignani G Ravetti JL Torre G Indiveri F CD8+CD28- T regulatory lymphocytes inhibiting T cell proliferative and cytotoxic functions infiltrate humancancers J Immunol 2007 179 4323 4334 17878327 10.4049/jimmunol.179.7.4323

[B22] Kriiger-Krasagakes S Krasagakis K Garbe C Schmitt E Hiuls C Blankensteinl T Diamantstein T Expression of interleukin 10 in human melanoma Br J Cancer 1994 70 1182 1185 10.1038/bjc.1994.469 7981073 PMC2033698

[B23] Gastl GA Abrams JS Nanus DM Oosterkamp R Silver J Liu F Chen M Albino AP Bander NH Interleukin-10 production by human carcinoma cell lines and its relationship to interleukin-6 expression Int J Cancer 1993 55 Suppl 1 96 101 8344757 10.1002/ijc.2910550118

[B24] Bouabe H Liu Y Moser M Boösl MR Heesemann J Novel highly sensitive IL-10–β-lactamase reporter mouse reveals cells of the innate immune system as a substantial source of IL-10 in vivo J Immunol 2011 187 3165 3176 10.4049/jimmunol.1101477 21844394

[B25] Halak BK Maguire HC Jr Lattime EC Tumor-induced interleukin-10 inhibits type 1 immune responses directed at atumor antigen as well as a non-tumor antigen present at the tumor site Cancer Res 1999 59 911 917 10029084

[B26] Vicari AP Chiodoni C Vaure C Aït-Yahia S Dercamp C Matsos F Reynard O Taverne C Merle P Colombo MP O’Garra A Trinchieri G Caux C Reversal of tumor-induced dendritic cell paralysis by CpG immunostimulatory oligonucleotide and anti–interleukin 10 receptor antibody J Exp Med 2002 196 Suppl 4 541 549 12186845 10.1084/jem.20020732 PMC2196048

[B27] Overwijk WW Restifo NP B16 as a mouse model for human melanoma Curr Protoc Immunol 2001 CHAPTER: Unit–201 John Wiley and Sons Inc 10.1002/0471142735.im2001s39 PMC2763508 18432774

[B28] Esquivel F Yewdell J Bennink J RMA/S cells present endogenously synthesized cytosolic proteins to class I-restricted cytotoxicT lymphocytes J Exp Med 1992 175 163 168 10.1084/jem.175.1.163 1309852 PMC2119061

[B29] Chiarle R Martinengo C Mastini C Ambrogio C D’Escamard V Forni G Inghirami G The anaplastic lymphoma kinase is an effective oncoantigen for lymphoma vaccination Nat Med 2008 14 Suppl 6 676 680 18469826 10.1038/nm1769

[B30] Wellings DA Atherton E Standard Fmoc protocols Methods Enzymol 1997 289 44 67 9353717 10.1016/s0076-6879(97)89043-x

[B31] Klein C Bueler H Mulligan RC Comparative analysis of genetically modified dendritic cells and tumor cells as therapeutic cancer vaccines J Exp Med 2000 191 1699 1708 10.1084/jem.191.10.1699 10811863 PMC2193145

[B32] Di Giacomo AM Danielli R Guidoboni M Calabrò L Carlucci D Miracco C Volterrani L Mazzei MA Biagioli M Altomonte M Maio M Therapeutic efficacy of ipilimumab, an anti-CTLA-4 monoclonal antibody, in patients with metastatic melanoma unresponsive to prior systemic treatments: clinical and immunological evidence from three patient cases Cancer Immunol Immunother 2009 58 1297 1306 10.1007/s00262-008-0642-y 19139884 PMC11030873

[B33] Seo N Hayakawa S Takigawa M Tokura Y Interleukin-10 expressed at early tumour sites induces subsequent generation ofCD4+ T-regulatory cells and systemic collapse of antitumour immunity Immunology 2001 103 449 457 10.1046/j.1365-2567.2001.01279.x 11529935 PMC1783257

[B34] Sheikh NA Petrylak D Kantoff PW Dela Rosa C Stewart FP Kuan LY Whitmore JB Trager JB Poehlein CH Frohlich MW Urdal DL Sipuleucel-T immune parameters correlate with survival: an analysis of the randomized phase 3 clinical trials in men with castration-resistant prostate cancer Cancer Immunol Immunother 2012 PMID: 22865266 [Epub ahead of print] 10.1007/s00262-012-1317-2 PMC3541926 22865266

[B35] Yang JC Melanoma vaccines Cancer J 2011 17 277 282 10.1097/PPO.0b013e3182325f72 21952276

[B36] Bergman PJ McKnight J Novosad A Sarah Charney S Farrelly J Craft D Wulderk M Jeffers Y Sadelain M Hohenhaus AE Segal N Gregor P Engelhorn M Riviere I Houghton AN Wolchok JD Long-term survival of dogs with advanced malignant melanoma after DNA vaccination with xenogeneic human tyrosinase: a phase I trial Clin Cancer Res 2003 9 1284 1290 12684396

[B37] Gold JS Ferrone CR JA G-P˜o Hawkins WG Dyall R Engelhorn ME Wolchok JD Lewis JJ Houghton AN A single heteroclitic epitope determines cancer immunity after xenogeneic DNA immunization against a tumor differentiation antigen J Immunol 2003 170 5188 5194 12734366 10.4049/jimmunol.170.10.5188

[B38] Iero M Filipazzi P Castelli C Belli F Valdagni R Parmiani G Patuzzo R Santinami M Rivoltini L Modified peptides in anti-cancer vaccines: are we eventuallyimproving anti-tumour immunity? Cancer Immunol Immunother 2009 58 1159 1167 10.1007/s00262-008-0610-6 18998128 PMC11030573

[B39] Filipazzi P Pilla L Mariani L Patuzzo R Castelli C Camisaschi C Maurichi A Cova A Rigamonti G Giardino F Di Florio A Asioli M Frati P Sovena G Squarcina P Maio M Danielli R Chiarion-Sileni V Villa A Lombardo C Tragni G Santinami M Parmiani G Rivoltini L Limited induction of tumor cross-reactive T cells without a measurable clinical benefit in early melanoma patients vaccinated with human leukocyte antigen class I-modified peptides Clin Cancer Res 2012 18 23 6485 6496 10.1158/1078-0432.CCR-12-1516 23032742

[B40] Moser M Leo O Key concepts in immunology Vaccine 2010 28S C2 C13 20713253 10.1016/j.vaccine.2010.07.022

[B41] Zepp F Principles of vaccine design—Lessons from nature Vaccine 2010 28S C14 C24 20713252 10.1016/j.vaccine.2010.07.020

[B42] Vergati M Intrivici C Huen N-Y Schlom J Tsang KY Strategies for cancer vaccine development J Biomed Biotechnol 2010 10.1155/2010/596432 PMC2914453 20706612

[B43] Dubensky TW Jr Reed SG Adjuvants for cancer vaccines Semin Immunol 2010 22 155 161 10.1016/j.smim.2010.04.007 20488726

[B44] Church SE Jensen SM Twitty CG Bahjat K Hu HM Urba WJ Fox BA Multiple vaccinations friend or foe Cancer J 2011 17 379 396 10.1097/PPO.0b013e3182346320 21952289 PMC3614402

[B45] Fujii H Arakawa A Kitoh A Miyara M Kato M Kore-eda S Sakaguchi S Miyachi Y Tanioka M Ono M Perturbations of both nonregulatory and regulatory FOXP3+ T cells in patients with malignant melanoma Br J Dermatol 2011 164 1052 1060 10.1111/j.1365-2133.2010.10199.x 21198537

[B46] Sakaguchi S Yamaguchi T Nomura T Ono M Regulatory T cells and immune tolerance Cell 2008 133 775 787 10.1016/j.cell.2008.05.009 18510923

[B47] Sakaguchi S Wing K Yamaguchi T Dynamics of peripheral tolerance and immune regulation mediated by Treg Eur J Immunol 2009 39 2331 2336 10.1002/eji.200939688 19662638

