# Supplementary material for: Comparative analysis of cancer vaccine settings for the selection of an effective protocol in mice
Source: J Transl Med. 2013 May 12;11:120. doi: 10.1186/1479-5876-11-120 (PMC3659084; doi:10.1186/1479-5876-11-120)
Supplement: Additional file 1 — Includes the Supplementary Figure 1 and the Supplementary Figure 2. [file 1479-5876-11-120-S1.pdf]

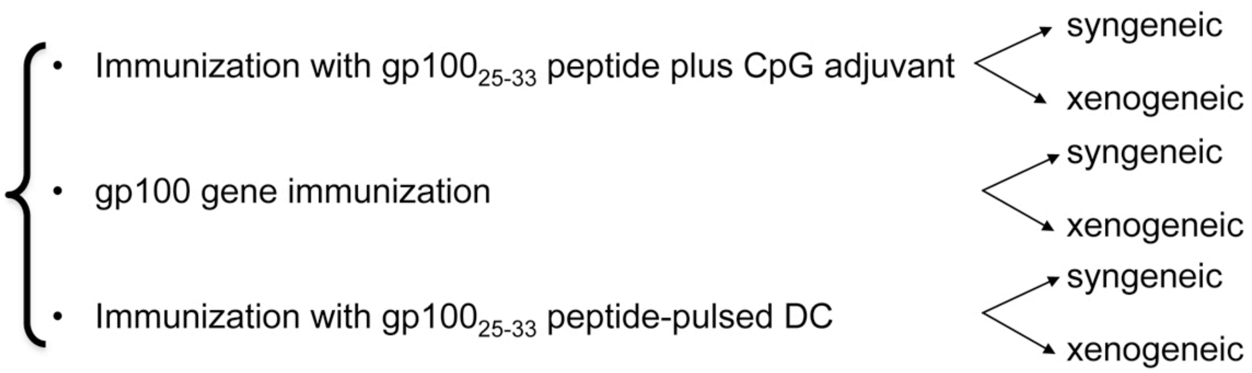

Supplementary Figure 1. **Summary of immunization protocols**.

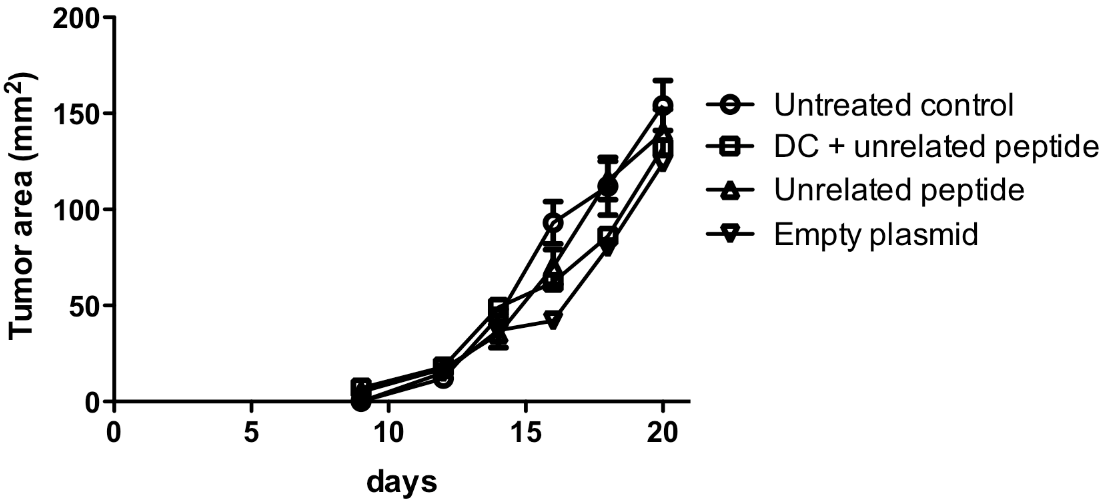

Supplementary Figure 2. Comparative analysis of the different control protocols for gp100 vaccination. Data, expressed as mean ± SD, refer to one of two separate experiments performed treating 4 mice per group. Identical results were obtained in the other experiment.
